# Point-of-Care Tests for Hepatitis B: An Overview

**DOI:** 10.3390/cells9102233

**Published:** 2020-10-02

**Authors:** Yinzong Xiao, Alexander J. Thompson, Jessica Howell

**Affiliations:** 1 Burnet Institute, 3004 Melbourne, VIC, Australia; yinzong.xiao@burnet.edu.au; 2 Department of Gastroenterology, St Vincent’s Hospital, 3065 Fitzroy, VIC, Australia; alexander.thompson@svha.org.au; 3 Faculty of Medicine, University of Melbourne, 3010 Parkville, VIC, Australia; 4 School of Public Health and Preventive Medicine, Monash University, 3004 Melbourne, VIC, Australia

**Keywords:** hepatitis B, point-of-care testing, hepatitis elimination, public health

## Abstract

Despite the heavy disease burden posed by hepatitis B, around 90% of people living with hepatitis B are not diagnosed globally. Many of the affected populations still have limited or no access to essential blood tests for hepatitis B. Compared to conventional blood tests which heavily rely on centralised laboratory facilities, point-of-care testing for hepatitis B has the potential to broaden testing access in low-resource settings and to engage hard-to-reach populations. Few hepatitis B point-of-care tests have been ratified for clinical use by international and regional regulatory bodies, and countries have been slow to adopt point-of-care testing into hepatitis B programs. This review presents currently available point-of-care tests for hepatitis B and their roles in the care cascade, reviewing evidence for testing performance, utility, acceptability, costs and cost-effectiveness when integrated into hepatitis B diagnosis and monitoring programs. We further discuss challenges and future directions in aspects of technology, implementation, and regulation when adopting point-of-care testing in hepatitis B programs.

## 1. Introduction

More than 257 million people, or 3.2% of the world’s population, are estimated to be living with chronic hepatitis B virus infection, with the greatest disease burden in low-resource countries in the Asia-Pacific and sub-Saharan Africa [1]. Without treatment, one in every four persons infected with chronic hepatitis B will develop liver cirrhosis over 20–30 years, and 2–5% of people with cirrhosis will develop liver cancer annually [2]. Globally, over 800,000 deaths annually are attributable to hepatitis B infection [1,3]. Most of this disease burden is preventable by appropriate guideline-based treatment [4,5,6,7,8].

Given the magnitude of the global public health burden from hepatitis B, the World Health Organization (WHO) has outlined ambitious hepatitis B elimination targets of a 65% reduction in mortality and a 90% reduction in incidence from baseline (2015) by 2030 [9]. However, current estimates suggest that we are a long way from achieving these goals unless investment and care cascade are scaled up [1,10]. The hepatitis B vaccination has greatly contributed to preventing transmission and reducing hepatitis B incidence globally; however, vaccination coverage is still suboptimal in resource-limited regions [11], and most countries in Africa have been unable to implement the hepatitis B birth-dose vaccine due to multiple logistical and cost barriers [12,13]. Meanwhile, for people who are already living with hepatitis B, receiving early diagnosis and clinical care is the key to reducing morbidity and mortality. However, in 2016, the WHO estimated that only 11% of people living with hepatitis B were diagnosed, among whom only 17% of those eligible were on treatment [1].

**Figure 1:**
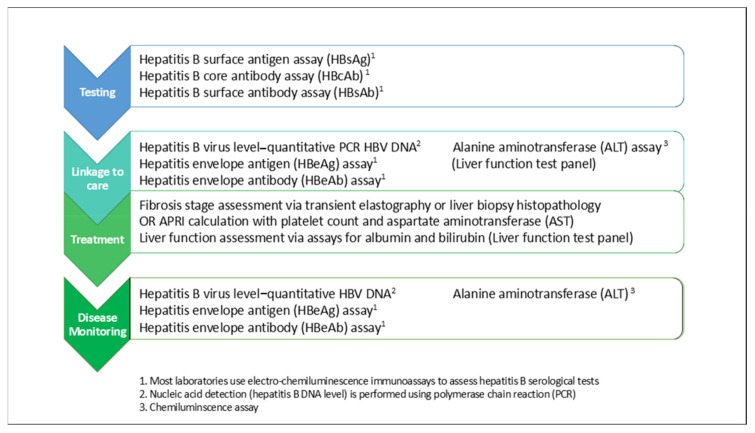
Cascade of care for hepatitis B and laboratory-based tests required for standard of care. Laboratory-based blood tests are required at every stage of the hepatitis B cascade of care for diagnosis, assessment of liver disease stage, treatment eligibility and long-term monitoring of disease progression. Diagnostic testing for hepatitis B involves detection of hepatitis B surface antigen (HBsAg) in blood, which indicates active infection with the virus. Standard laboratory electro-chemiluminescence immunoassay-based HBsAg testing is performed on serum or plasma samples derived from whole blood. If active infection is confirmed, subsequent blood tests are performed to determine the stage of disease and need for treatment, including a hepatitis B virus (HBV) polymerase chain reaction (PCR)-based quantitative DNA level or viral load, a hepatitis B eAg and eAb assay and liver function tests to determine whether an elevated aminotransferase (ALT) indicative of liver inflammation or other signs of impaired liver function are present. Further assessment for the presence of liver fibrosis and cirrhosis is also required, most commonly by transient elastography and/or liver biopsy. All patients irrespective of treatment require ongoing disease monitoring, including at minimum an HBV DNA level, HBeAg and HBeAb (if not already seroconverted from HBeAg positive to HBeAb positive) and ALT levels every 3–6 months.

The hepatitis B cascade of care involves multiple steps: screening, diagnosis, linkage to care, assessment of liver disease stage and treatment eligibility, then treatment and/or monitoring, including surveillance for hepatocellular carcinoma (HCC) (Figure 1). Laboratory blood tests are required at every step of the care cascade, including blood tests for hepatitis B serology, quantitative hepatitis B virus (HBV) DNA level by polymerase chain reaction (PCR) and liver function tests (Figure 1). These tests require laboratory resourcing, technology and expertise beyond existing peripheral laboratory capabilities in many low-resource and geographically isolated regions [14,15,16]. In many countries, laboratory services are centralised due to high costs and limited skilled technician capacity; however, transport of blood samples from regional to centralised laboratories presents its own challenges in geographically isolated or insecure regions, particularly if cold chain supply must be preserved [14]. Cost is another major limitation: Price reductions for diagnostics have fallen slowly over time compared with medication costs, and hepatitis B diagnostic tests cost more than therapy in many low-income countries [16,17]. Moreover, the requirement for lifelong monitoring for most people living with hepatitis B that involves regular blood tests [4,5], combined with barriers to timely healthcare access such as hepatitis B-related stigma [18,19], healthcare costs for users and providers [20] and the logistics of accessing consistent, high-quality, affordable healthcare services in a timely manner are major barriers for people to receive guideline-based care [16]. These barriers lead to significant attrition from every step of the hepatitis B care cascade over time, and those lost from care represent missed opportunities for treatment and liver cancer prevention [16,21].

**Figure 2:**
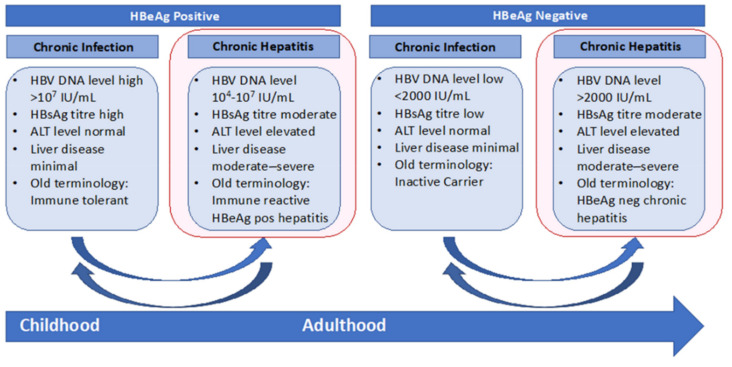
Natural history and clinical characteristics of the phases of chronic hepatitis B infection. Current international hepatitis B management guidelines have different criteria for initiating hepatitis B treatment; however, all restrict nucleos(t)ide therapy to those with at least moderate liver fibrosis and/or inflammation and a high viral load.

Point-of-care tests (POCs; also known as rapid diagnostic tests, RDTs) are simplified versions of laboratory-based tests that have the potential to circumvent major barriers people face to accessing hepatitis B blood-based testing in various settings. POCs usually require small amounts of body fluids (for example, a finger-prick blood sample or oral swab), short turn-around time, and are generally easy to use with minimal required training and therefore can be provided to people in a variety of community and outreach settings by a broad range of trained workers [22] and are scalable to rapidly reach large populations as has been seen with the highly successful Egyptian national hepatitis C screening program [23]. The simple collection process (finger-prick or mouth swab) is also highly acceptable, feasible and attractive to people undergoing testing [22,24,25]. A key benefit of POCs in the field of hepatitis B is to engage hard-to-reach communities for testing, such as using HBsAg POC tests for hepatitis B screening in remote areas, or harm reduction programs [24,25,26,27]. POCs also have great potential for retaining patients in care when used in the community for chronic hepatitis B stage evaluation and disease monitoring [26,27]. Figure 2 outlines the key phases of disease in chronic hepatitis B infection and the indicators for blood testing in each stage.

The WHO recommends that an ideal POC test needs to meet the ASSURED criteria of being “affordable, sensitive, specific, user-friendly, rapid and robust, equipment-free and deliverable to end-users” [28]. Since 1998, the WHO has implemented an evaluation and performance assessment program for all POCs in viral hepatitis to report on accepted quality parameters for widespread clinical use [29]. Many POCs have been developed in the field of hepatitis B, particularly for screening and diagnosis; however, only three POCs for detecting HBsAg have been prequalified by the WHO [29]. There is currently a lack of POCs for hepatitis B stage assessment or monitoring that have been endorsed to use by the WHO; however, several novel tests are now in clinical trials that may fill this important care delivery gap. Typically, POC tests have lower accuracy than traditional laboratory-based tests, but they facilitate the triage of people who require more complex and expensive laboratory assays to confirm a positive POC test result and thereby reduce costs. Regulatory and economic constraints are additional barriers to transferring POCs to field use. In different settings, they therefore require a comprehensive appraisal of factors including testing performance, feasibility (such as storage requirements, power supply), acceptability and cost-effectiveness when using POCs to scale up access to hepatitis B diagnosis and management under real-life conditions.

In this review, we outline the accuracy of available POC tests for hepatitis B and explore the evidence for utility and cost-effectiveness when integrated into hepatitis B diagnosis and monitoring programs. We also describe future technologies and explore how POC tests might best be used to achieve WHO 2030 hepatitis B elimination goals.

## 2. POC Tests for Hepatitis B and Their Clinical Performance

Practically, the three key clinical requirements for POC hepatitis B assays in the field are for diagnosis of current infection, determining treatment eligibility and also monitoring, as well as diagnosis of hepatitis B immunity and the need for hepatitis B vaccination in the uninfected (Figure 2).

### 2.1. Diagnosis of Current Hepatitis B Infection

Detection of hepatitis B surface antigen (HBsAg) is the primary step to diagnose current hepatitis B infection, and multiple HBsAg POCs are commercially available. Most are qualitative lateral-flow chromatographic immunoassays which are one-step, easy to use, can be used with a variety of different specimens (whole blood, serum and plasma) and provide rapid semiquantitative visible results (usually within 15–30 min). To date, three HBsAg rapid tests (Determine HBsAg 2, Alere Medical Co. Ltd, Chiba-ken, Japan; VIKIA HBsAg, bioMérieux SA, Marcy-l’Étoile, France; and SD Bioline WB, Abbott Diagnostics Korea Inc. Giheung-gu, Republic of Korea) have met WHO prequalification criteria [29], with multiple studies showing their high accuracy for determining HBsAg positivity in various populations, particularly in moderate–high-prevalence populations (Table S1).

Determine HBsAg POC test is the one of the most widely-used HBsAg POC tests [30] with the most published data on clinical performance. A 2017 meta-analysis [31] including 9 studies with 7730 samples showed a pooled sensitivity of 90.8% and specificity of 99.1% using Determine. Though most studies [32,33,34,35,36,37,38] showed high clinical sensitivity of 89–100% in the general population, the reported sensitivity varied widely in HIV-infected populations (56–100%) [39,40,41,42,43,44]. The cause of the reported lower sensitivity in HIV-coinfected populations [39,40,43,44] is unclear, but potential reasons may include the cross reaction of HIV-reverse transcriptase inhibitors and hepatitis B virus, a higher rate of occult hepatitis B infection in early HIV cohorts, a higher reported rate of HBsAg loss in both untreated and treated HIV-infected populations and the use of tenofovir-based HIV regimens that effectively suppress hepatitis B virus DNA levels and a large decline in HBsAg titres [31,45,46]. SD Bioline HBsAg [38,47,48,49] and VIKIA HBsAg POC test [32,33,38,50,51] have also been shown to have good sensitivity (above 90%) and excellent specificity (above 99%) in general populations; however, lower sensitivity was also reported in HIV-infected populations [40].

A common application of these HBsAg POCs is to measure seroprevalence in general or specific subpopulations in low-resource settings [52,53,54,55,56]. They have also been used in mass screening programs for hepatitis B in both community outreach [24,57] and health-facility-based screening [52,53,58,59,60,61,62] in low-resource settings and shown great public health benefits. For example, in a community-based outreach screening program conducted in 75 camps in Southern India [63], the “screen and vaccinate/linkage to care” strategy led to over 7700 vaccinations in the camps and 162 people with high viral load getting treatment. The program increased the accessibility of hepatitis B diagnostic testing in a low-resource setting, and the timely results of POCs contributed to people’s engagement in post screening interventions [63]. The HBsAg POCs were also used in programs to engage hard-to-reach populations such as people who inject drugs, sex workers [64], disadvantaged groups or some ethnic groups [65,66] by providing self-testing, community or health-facility-based testing services. In a randomised control study conducted in a clinic engaging mostly African immigrants in France [65], people without health cover attending a clinic were provided free testing for hepatitis B, hepatitis C and HIV using either POCs or prescriptions of testing at a pathologist; a higher rate of testing and linkage to care was observed among people allocated to receive POCs. However, another multicentre randomised control study in France [66] found no difference in effectiveness of linkage to care using the approach of an HBsAg rapid test plus a standard lab-based confirmatory serology test versus lab-based standard serology in five clinics. In this study, it was described that participants received testing results via mail or phone call, but it was unclear whether participants received testing results at the same visit if they were in the POC testing group [66].

Other than the WHO-prequalified HBsAg POCs, emerging new brands of HBsAg have been reported in field studies including the DRW-HBsAg assay, Diagnostics for the Real World Ltd., (CE-marked) [35,67], first response HBsAg card test, Premier Medical Corporation [68] (CE-marked), NaoSign(R) HBs POC strips, Bioland [69], and One Step HBsAg test, General Biologicals Corporation [70,71] (non-exhaustive list). A study in Mongolia [72] which tested 19 commercially available HBsAg POCs using a serum sample showed the average sensitivity and specificity being 100% and 99%, respectively. Whilst most HBsAg POCs of various brands have shown promising clinical performance [35,38,67,70,73], available validation data are limited and further studies with large sample size and in diverse populations including different ethnicity and hepatitis B prevalence populations and people living with HIV are needed.

Multiplex diagnostic POCs can be highly attractive for low–middle-resource settings with the capacity to detect multiple pathogens using a single testing strip. Some multiplex POCs which detect HBsAg are commercially available and some are CE-marked (HBsAg/HCV/HIV/Syphilis Combo Test, Euro Genomas; HBsAg and HCV Combo Test, Euro Genomas; Artron Detect 3 HIV/HCV/HBV Combo, Artron Laboratories; HIV, HBsAg and HCV Rapid Test, Maternova Inc., Providence, RI, USA), but none have been listed by WHO prequalification [16,29,74]. Accuracy of HBsAg detection using multiplex has been shown to be high [75], but limited clinical validation data are available.

Innovations in sampling technique have provided more convenient specimen collection methods, such as using oral fluid as specimen collected by an oral swab [25,48,51]. The simplified process was highly acceptable to individuals [25,48], but testing accuracy is a challenge to overcome [48], and it may additionally require trained technicians or lab-based enzyme immunoassays or equipment for sample preparation such as requiring a centrifuge for target analyte separation [51]. Future development needs to consider combining sample preparation steps together with detection and readout into one single device, without sacrificing testing accuracy.

Although many studies of different HBsAg POCs showed very good sensitivity [31,38,73], false negativity is still among the biggest concerns: Around one in ten negative test results on average could be HBsAg positive [31]. Most cases with false negative results were reported to have low titres of HBsAg, such as in studies using Determine/Vikia HBsAg POCs, where most false negative cases had HBsAg titres lower than 30 IU/mL [32,33]. As HBsAg level does not correlate with severity of liver damage, there is a chance that people with advanced liver disease may be missed. Other potential factors affecting the accuracy of HBsAg POCs may include HBV DNA level, different genotypes, co-infection with hepatitis C or HIV and hepatitis B variants with S gene mutations that are not detected by the POC HBsAg test [31,33,76,77]. As only a few studies have obtained comprehensive serological and genetic profiles of false negative cases, more data are needed to explore these associations and determine the implications for clinical practice. Specimen type is unlikely to affect the efficacy of HBsAg POC tests: A meta-analysis showed similar pooled sensitivity of studies using whole blood sample compared to plasma or serum [31], and studies evaluating HBsAg POCs using capillary whole blood collected by finger-prick all showed reasonably high sensitivity (88–90%) [32,44,78]. In practice, there is no absolute cut-off for testing performance when choosing POCs for hepatitis B programs, and the increased access to testing might mitigate the harm caused by reduced accuracy; however, sensitive POCs that have been validated in similar contexts to their planned use should be prioritised [79].

### 2.2. Diagnosis of Hepatitis B Immunity

Hepatitis B surface antibody (anti-HBs) is the key marker to determine an individual’s immunity status to hepatitis B virus and triage the need for vaccination. A few anti-HBs POCs are commercially available, but most have poor reported sensitivity ranging from 20% to 70% [33,80,81,82]. One study reported a sensitivity of 91.8% using an anti-HBs rapid test card among 1272 samples [70]; however, these findings require further validation. A study [66] showed using HBsAg/ anti-HBs POCs was not effective in increasing vaccination rate due to poor sensitivity of anti-HBs POC and high reliance on confirmatory enzyme immunoassay. Though a POC test for anti-HBs can help with triage vaccination need, given hepatitis B vaccination is relatively cheap, context-specific cost-effectiveness analyses would be needed to determine settings where the use of POCs of anti-HBs would be cost-effective.

### 2.3. Chronic Hepatitis B Care: Treatment Eligibility and Long-Term monitoring

Treatment decisions in hepatitis B are guided by patient age, hepatitis B DNA viral load and the degree of liver inflammation and fibrosis, as measured by alanine aminotransferase (ALT) levels and either transient elastography or liver biopsy, respectively [4,5,83]. However, there are few POC tests currently available for these parameters, and none have been widely validated and WHO prequalification approved.

#### 2.3.1. Hepatitis B DNA Quantification

Hepatitis B virus (HBV) DNA level is the critical indicator when deciding an individual’s management plan as per clinical guidelines. Polymerase chain reaction (PCR) platforms for nucleic acid detection are still the main technique of quantitative assessment HBV DNA levels; conventional PCR platforms are usually built in laboratories and require high manual input and pose barriers for accessibility in remote areas and other resource-limited areas areas [12]. A rapid molecular test, Xpert^®^ HBV Viral Load (Cepheid Inc., Sunnyvale, CA, USA, CE-marked, approved by American FDA and TGA in Australia), is commercially available for HBV DNA quantification that provides test results in less than one hour [84,85]. The test is a cartridge-based, real-time PCR assay which is run on the GeneXpert instrument, a molecular diagnostic platform. The processing unit of the system is around the size of a coffee machine, and it also runs a range of other rapid molecular tests such as WHO prequalified Xpert HCV viral load, HIV-1 Qual and HIV-1 Viral Load tests [29], which poses an opportunity for hepatitis B viral load test to be adopted in areas with existing platforms at a low additional cost. So far, limited data are available on the analytical performance of the assay [84,85]. Two recent studies [84] using serum samples showed a good correlation between HBV DNA quantification by using Xpert HBV viral load assay with the results of the laboratory reference assay; they also have a low limit of detection (LOD) of 7.5 IU/mL, which is similar to most commonly used HBV DNA platforms (usually with LOD of 10IU/mL) [84]. In practice, Xpert testing for HBV DNA led to a faster workflow with a mean time to result being 6–8 h, which provided a near-POC solution [84,86]. However, as a new unit, GeneXpert facilities are still expensive; the operation requires uninterrupted power supply, as well as technician training and skills for system running, services and reagent maintenance.

#### 2.3.2. HBeAg/Anti-HBe

Hepatitis E antigen (HBeAg) is a key indicator to determine phase of chronic hepatitis B infection (Figure 2), treatment initiation and is used as a surrogate of HBV DNA measurement for evaluating risks of maternal-to-child-transmission [2,4,83,87]. Several HBeAg POCs are commercially available; however, published data show the accuracy of HBeAg POCs has a wide range, with sensitivity of 30–82% and specificity of 67–100% [33,80,88]. Similarly, Anti-HBe POCs are reported to have poor sensitivity but excellent specificity in studies [33,81]. Given the high costs and challenges in accessing HBV DNA testing in low-resource settings, the WHO recommends HBeAg to triage treatment [83,89,90]; therefore, the low testing accuracy of HBeAg POCs is an urgent issue to be addressed.

#### 2.3.3. Novel Biomarkers

Novel serum biomarkers such as HBV core antigen (HBcrAg) have been shown to correlate with serum HBV DNA levels and intrahepatic cccDNA levels, a marker of hepatitis B-related HCC risk, and have therefore been explored as a potential indicator for treatment determination, off-therapy virologic suppression and HCC risk evaluation [91,92]. However, there is no rapid lateral flow assay for HBcrAg so far. Another novel biomarker, serum HBV RNA [93], has been shown to be positively correlated with HBV DNA level, and levels were higher than HBV DNA in patients on nucleos(t)ide analogues and can thus be a potential marker for off-therapy HBV suppression. However, its clinical predictive utility is not yet well defined, and the measurement of serum HBV RNA presents its own challenges even in routine lab-based testing; thus, further studies are needed to guide defining the clinical role and development of HBV RNA POCs.

#### 2.3.4. ALT and Assessment of Liver Fibrosis

ALT is part of the liver function test assay panel and is a key marker of liver inflammation, used to determine hepatitis B treatment eligibility [4,5,83] (Figure 2). ALT has been proposed as an indicator for treatment in people with positive HBsAg in low-resource settings where HBV DNA testing is unavailable. A semiquantitative POC using ALT 40U/L as the cut-off (BioPoint^®^ALT-1) has been developed and manufacturer data suggest a high sensitivity of 94% and specificity of 85% [94]. Several serological biomarkers have been combined in algorithms to offer indirect non-invasive assessment of liver fibrosis and have been validated to varying degrees in hepatitis B populations, such as the AST to platelet ratio index (APRI) and Fibrosis 4 index (FIB-4) [95,96]. These indices require quantitative testing results of AST, platelets with or without ALT, unfortunately none of which are currently available in a POC test format.

#### 2.3.5. Dried Blood Spot

Dried blood spot (DBS), while not a POC test, is a sampling method which offers viable solutions for mass screening or testing in low-resource settings where testing capacity or access are limited. In practice, a single finger-prick blood sample is applied to a chemically modified paper card which collects and store serological markers and nucleic acid; specimens obtained in the field can be transported to a laboratory at ambient temperatures, where the blood sample is processed following a DBS protocol and tested using immunoassays or molecular techniques [83,97]. DBS samples have a relatively long shelf-life at ambient temperature without sample degradation [98], which is attractive for regions that are geographically isolated or have varying security situations precluding rapid transport to a central laboratory. This method is now recommended by the 2017 WHO hepatitis B testing guidelines [83] in settings where no access to venous blood sampling or quality-assured testing assays is available. DBS testing has been used to detect HBsAg, HBeAg, anti-HBc, HBV DNA and even for viral genotyping [99,100]. A meta-analysis [101] evaluating DBS for HBV DNA quantification showed pooled sensitivity of 95% (83–99%) and specificity of 99% (53–100%); however, most of the included studies used cold chain to store samples, which might limit the generalisation of the accuracy estimates in field conditions. Although DBS testing increases testing access in low-resource and geographically isolated settings, it still requires high technical expertise and standard laboratory assays that may not be routinely available.

## 3. Cost-Effectiveness of Using POCs for Hepatitis B

Cost-effectiveness and affordability are key considerations when adopting POC tests in hepatitis B programs. Quoted costs of lateral flow-based HBsAg POCs are generally lower than laboratory-based immunoassays, with the estimated procurement costs being US$0.2–0.95 and US$0.4 to 2.8 per test, respectively [72,83]. Conventional lab-based testing usually requires additional costs such as a reading machine, professional laboratory staff and technical training; therefore, the total costs for testing are often much higher than using POCs in low-resource settings.

Multiplex POC testing is expected to be cheaper than multiple POC tests. For example, the manufacturing costs of a HIV/HCV/HBsAg POC is around US$1 [102]; thus, using multiplex in high-risk populations who require broad spectrum pathogen screening is expected to be resource-saving. Costs for conventional hepatitis nucleic acid testing are estimated to range from US$30 to 120 [15], and the cost can be up to US$400 per assay in resource-limited countries and regions [12]. In 2018, a viral load testing program was introduced in sub-Saharan African countries to access an integrated molecular diagnostics instrument (Hologic Panther system), at an all-inclusive ceiling price of US$12 per patient sample [103]. The Foundation for Innovative New Diagnostics (FIND) has negotiated the price of Xpert HBV viral load assay for 145 developing countries, and it costs US$14.9 per cartridge excluding shipment; however, the testing instrument costs between US$11,530 to US$64,350 depending on the throughput capacity of the processing unit [104,105].

However, these costs may still be higher than what programs could afford in some settings. In addition, due to reduced accuracy compared with standard assays, diagnostic POC testing is often used as a screening tool to triage those requiring more expensive laboratory-based testing confirmation [15], which means many of the costs for centralised laboratory services are only partially offset by POC test use. While novel POC testing may have increased testing performance, costs usually fall slowly due to patent protection laws [16]. Even for countries that could afford these POCs, it may cost more than lab-based testing where well-established laboratory services are available; therefore, the main demand for POCs is limited to self-testing or outreach programs to improve testing uptake.

The cost-effectiveness of using POCs for hepatitis B can therefore be different in different settings. Using HBsAg POCs as a screening tool was found to be cost-effective in community-based approach in HBV-endemic but low-resource settings. Nayagam et al. [106] assessed a community-based HBV screening and treating program in The Gambia where HBsAg POCs were provided to adult participants door-to-door at a total screening cost of US$7.4 per person. The program was found to be highly cost-effective, with an ICER of US$540 per DALY averted compared to status quo where no publicly provided HBV screening or treatment was available. Integrating low-cost HBV POCs into existed healthcare services such as antenatal screening [52,58,107], blood donor screening [62] and HIV clinics [59,60,61] can be another solution to achieve scale-up of HBV testing [108,109]. Zhang et al. [109] showed the integration of HBV screening within the existing antenatal care in Cambodia was highly cost-effective. In their model, the unit cost of HBsAg and DNA test (estimated to be US$1 and US$30) was one of the key parameters driving cost-effectiveness; in such cases, cheap POCs could potentially improve the cost-effectiveness of such an integration program even further. Studies in low HBV endemicity countries showed programs offering hepatitis B screening followed by vaccination or linkage to clinical care among people with increased risks are likely to be cost-effective [110]; however, there is a lack of programs adopting POCs in hepatitis B screening strategy, and thus, a lack of evidence suggesting economic impacts using POCs for hepatitis B in populations who have regular access to healthcare services. However, a few studies have shown rapid hepatitis C or HIV testing nested in harm reduction programs or among priority populations can be cost-effective [111,112,113]. More evidence on using POCs in HBV screening or monitoring programs in the field is needed, especially covering the implementation costs and the effects of broader testing access compared to standard testing services or no testing services (where there being no access to testing is the current practice).

## 4. Utility and Acceptability of POCs for Hepatitis B

Whilst POC testing theoretically circumvents many test access barriers, acceptability from targeted population remains a key determinant of successful implementation of hepatitis B programs. However, limited data are available on the satisfaction appraisal from users and stakeholders.

In general, POCs are highly acceptable to customers due to their easy-to-use nature, short turnaround time, minimal bio sample requirement and provision of testing capacity to familiar staff in contexts where people want to be tested [114]. In a survey conducted among implementers and users of hep B and C testing services from 43 countries, almost half of respondents from low- and middle-income countries preferred a POC test method using capillary whole blood [83]. While there is no agreement on what accuracy would be considered acceptable, half of the respondents would accept an assay with a minimal sensitivity of 95% [83].

Acceptability of rapid testing for hepatitis B or other blood-borne viruses and sexual transmitted diseases can be varied in different populations. A survey done in a prison setting showed HCV POCs were highly accepted [115]. Another study showed that people may find it stressful when testing HIV, HCV and syphilis using a POC test [116]. When using POCs for blood-borne virus screening in public events or community outreach programs, the acceptance rate varied widely in customers with different socioeconomic status, ethnic or geographic backgrounds [63,117,118]. In health facility settings, healthcare providers find POCs generally speed up decision making and improve patients’ compliance with chronic management plans requiring repeat testing over time [30]; however, there are also general concerns such as suboptimal testing accuracy and increased workload for healthcare workers [119].

Other than getting tested from healthcare providers or trained personnel, POCs also have the potential to be a self-testing tool with universal access. In some countries, rapid tests for hepatitis B and/or HIV and hepatitis C can be purchased online or over the counter. While self-testing offers a confidential testing solution for customers, a standard approach will be needed to ensure that people having accessible pre- and post-counselling, as well as pathways of linkage to care.

## 5. Limitations

A general limitation of POCs for hepatitis B is reduced accuracy compared to standard lab-based testing. There are also specific limitations for individual POC tests which were highlighted above (Section 2), such as HBsAg POC tests which were shown to have varied sensitivity in HIV-infected populations. In addition, there is still a lack of POC tests for liver cirrhosis and HBV DNA levels required to determine treatment eligibility for patients with hepatitis B.

There are also limitations in the aspects of regulatory process, procurement and storage management for POC tests, as well as costs when implementing POCs for hepatitis B in different settings. The WHO prequalification process for in vitro diagnostic tests for diseases with a high individual or public health risk, including hepatitis B, assesses both the test’s performance and manufacturing quality. For countries without regulatory procedures in place, this provides a thorough review of potential diagnostic tests they could select based on specific needs; however, the process of getting prequalified approval by the WHO can be slow. For low-resource settings, stock-out and supply issues can be a barrier for use of POC tests; lack of scale may also mean they can be more expensive than high throughput assays in some settings; testing accuracy as well as instrument maintenance can be impacted by extreme weather conditions (heat, humidity) in the field; novel testing platforms such as GeneXpert can still be expensive, and the use of the instruments can be subject to field conditions such as power supply. On the other side, for high-income countries, a main challenge for the introduction and implementation of POC tests is the regulatory and reimbursement approval process for new diagnostics, which require demonstration of analytic and clinical validity, as well as clinical usefulness and cost-effectiveness data. As an example, the FDA regulatory process can be long and expensive [120], and the return for investment in high-income countries where POC tests will compete for market with standard diagnostic pathways can be challenging.

## 6. Future Directions

### 6.1. Technology Needs

Increasing testing accuracy is the major challenge for POC tests that are already compact and easy to use. When developing POC diagnostics, features targeting resource-limited settings without basic infrastructures or cold chain need to be included; tests with high quality need to be validated across populations and specimen type. Rapid affordable serology tests of high accuracy for novel biomarkers which could be alternatives for molecular testing are a major need. Technology is needed to integrate convenient sampling and specimen preparation into a one-step testing assay. Inter-user variability is another challenge to address if POC tests require technique training or multiple steps; a standard protocol or mobile apps can be used to overcome this problem where suitable. Miniaturisation of testing instruments is the trend, especially for instruments that could perform molecular analysis such as portable hand-held devices, without sacrificing testing accuracy.

### 6.2. Implementation Approach

Dry blood spot kits for HIV and hepatitis C can already be ordered online to be sent to a home address as a private way to test for infection [121]. Faecal occult blood tests are mailed out to all older adults in some regions as a public health initiative to screen for bowel cancer [122]. A similar approach could be evaluated to screen for hepatitis B among populations that are disengaged from traditional health services. In resource-limited settings, adopting hepatitis B testing in existing platforms or programs can be more cost-effective than starting a new initiative [109]. Mobile phone technology has the potential to be used for screening and monitoring health conditions [123]. Mobile phones are now being used around the world for contact tracing for SARS-CoV-2, an approach that is immediately applicable to hepatitis B. Recently, Google searches for anosmia have been linked to the epidemiology of SARS-CoV-2 [124].

### 6.3. Regulatory Approval

There is a need for the streamlining of regulatory and reimbursement approval processes in high-income countries where the traditional approval process is expensive and slow, particularly for POC diagnostics suitable for use as public health tools to promote the engagement of marginalised individuals, including people who inject drugs, migrants and culturally and linguistically diverse communities affected by hepatitis B. In low- and middle-income countries where regulatory processes can be less demanding, the key is to ensure the quality and performance of tests as they come to market. More than 60 products have been prequalified since the WHO prequalification process started in 2010 [29]. It has been proposed that a model list of essential diagnostics be developed, comparable with the model list of essential medicines maintained by the WHO. Such a list would help in the selection of diagnostic methods and would facilitate improvements in the regulation and affordability of in vitro diagnostic tests and in training in their use.

## 7. Conclusions

The WHO has set ambitious goals for the elimination of hepatitis B as a public health threat by 2030. Birth dose vaccination is the most important public health intervention to reduce incidence and will also reduce mortality level long term. For the individual already infected with hepatitis B, the key to preventing liver-related harm is the maintenance of sustained viral suppression. This requires diagnosis and linkage to care; in some people, antiviral therapy will be necessary. Hepatitis B is typically asymptomatic until advanced disease has developed. Therefore, screening is required. The risk factors and epidemiology of hepatitis B are well described, but screening rates are suboptimal and often occur in the context of opportunistic doctor–patient consultations following presentation with an unrelated problem. Testing typically involves venesection followed by centralised testing in a laboratory with batch processing and automation to improve efficiency. This system works well for the engaged individual being cared for by a motivated health care practitioner. However, even in high-income countries, up to 80% of infected patients remain unaware of their infection [125]. Thus, there is a need to scale up screening for hepatitis B in high-risk populations, and a need to reconsider current models of care for screening. Now that hepatitis B treatment is cheap, safe and highly effective and durable, there is an urgent need to reconsider a public health approach to the management of hepatitis B. Point-of-care tests provide a tool for mass screening in community settings. They also provide the opportunity to reduce the care cascade to one of same-day “test and treat”. The effective employment of such strategies will be necessary for achievement of WHO elimination goals.

## Supplementary Materials

The following are available online at https://www.mdpi.com/2073-4409/9/10/2233/s1.
