# Supplementary material for: Point-of-Care Tests for Hepatitis B: An Overview
Source: Cells. 2020 Oct 2;9(10):2233. doi: 10.3390/cells9102233 (PMC7650625; doi:10.3390/cells9102233)
Supplement: Supplementary file 1 [file cells-09-02233-s001.pdf]

Supplementary Materials: The following are available online at www.mdpi.com/xxx/s1,

Table 1. Studies on HBsAg rapid tests (Determine™, VIKIA®, and SD Bioline).

Study	**Manufacturer**	**Location**	**setting**	**Population**	**Sample size**	**Specimen used**	**Sensitivity (95% CI)**	**Specificity (95% CI)**
Determine™ (WHO PQ listed in September 2019)								
Njai 2015 [33]	Alere, Waltham, MA, USA	Gambia	Field	General population	773	whole blood (finger prick)	88.5%	100%
			Lab	Hep B patients	178	Serum	95.3% (90.5%–98.1%)	93.3% (77.9%–99.2%)
Dembele 2020 [39]	Alere, Ireland	Abidjan, Ivory Coast	Lab	Plasma sample from biobank and whole blood sample from blood donors	1104	Plasma, whole blood	100% (99.2–100%)	100% (99.2%–100%)
Hirzel 2015 [129]	Iverness Biomedical Innovations, Germany	Switzerland	Lab	Clinical plasma samples with S gene mutated HBV and samples without HBsAg	70	Plasma	100%	100%
Bottero 2013 [34]	Inverness Biomedical Innovations, Germany	Paris, France	Field	Individuals attending 10 health centres	2472	whole blood (venepuncture)	93.6% (lower 97.5%CI: 80.7%)	100% (lower 97.5%CI: 99.8%)
Lien 2000 [35]	Abbott Laboratories	Vietnam	Lab	Patients attending different hospitals and disease centres	328	sera/plasma/whole blood (venepuncture)	100% (97%–100%)	100% (98%–100%)
Lin 2008 [36]	Inverness Medical Innovations	China		Blood donors and clinical specimen	671		98.92% (97.44%–100%)	100% (100%–100%)
		Guinea					94.44% (91.1%–97.8%)	100% (100%–100%)
Randrianirina 2008 [37]	Abbott Laboratories,	Madagascar	Lab	Stored serum samples (91 HBsAg+, 109 HBsAg−)	200	Serum	97.8% (94.2%–100%)	100% (99.2%–100%)
Nyirenda 2008 [40]	Abbott Laboratories, IL, USA	Malawi	Field	Hospital patients (17.5% were HBV positive, 78% were HIV positive; 16% were HBV/HIV co-infection)	194	plasma	56.00%	69%

Geretti 2010 [41]	Germany Inverness Medical, United Kingdom	Ghana	Lab	HIV positive population	838	serum, plasma	69.3% (61.6%–76.9%)	100% (100%–100%)
Davis 2010 [42]	Inverness Medical, Japan	Malawi	Lab	HIV positive population (Malawi sample tested in the UK)	75	sera	100% (86%–100%)	100% (93%–100%)
Franzeck 2013 [43]	Alere Inc., USA	Tanzania	Lab assessed	HIV positive population (ART naïve) (9.2% HBsAg detected)	272	plasma	96% (82.8%–99.6%)	100% (98.9%–100%)
Hoffman 2012 [44]	Abbott Laboratories, Brazil	Soweto, South Africa	Lab assessed	HIV positive population attending antenatal clinics or primary care (ART naïve) (4.2% HBsAg positive)	973	unclear	75% (58.5%–86.8%)	99.6% (98.8%–99.9%)
Chisenga 2018 [45]	Alere Inc., MA, USA	Zambia	Field	HIV-infected population at 2 urban clinics; 16% were HBsAg positive	412	whole blood (finger prick)	87.9% (77.5%–94.6%)	99.7% (98.4%–100%)
Chotun 2017 [79]	Alere Inc., MA, USA	Cape Town, South Africa	Field	HIV-uninfected pregnant women attending an antenatal clinic (6/134 (4.5%) tested HBsAg positive)	134	whole blood (finger prick)	unclear	100%
Paul 2018 [38]	Alere Inc., Japan	Bangladesh	Lab	Children and mothers from a sero-survey with positive HBV	34	Whole blood (venepuncture)	91.2% (76.6%–98.1%)	100% (99.9%–100%)
VIKIA® HBsAg (WHO PQ listed in Jul 2018, to be discontinued[106])								
Geretti 2010[41]	BioMerieux, UK	Ghana	Lab	HIV positive population	838	serum, plasma	70.7% (63.2%–78.3%)	100% (100%–100%)
Bottero 2013[34]	Biomerieux, France	Paris, France	Field	Individuals attending 10 health centres	3928	whole blood (venepuncture)	96.5% (Lower 97.5%CI: 89%)	99.9% (Lower 97.5%CI: 99.8%)
Hirzel 2015 [129]	BioMérieux, France	Switzerland	Lab	Clinical plasma samples with HBV containing HBsAg mutations of without HBsAg	70	Plasma	100%	100%

Njai 2015[33]	BioMérieux, France	Gambia	Field	General population	476	whole blood (finger prick)	90% (79.5%–96.2%)	99.8% (98.7%–100%)
Cruz 2015[52]	Biomérieux, France	Brazil	Lab assessed	Samples from field studies with different risks of HBV	1503	whole blood (venepuncture) / serum/ saliva	93.82 % (89.21–96.87)	99.92 % (99.58–100.00)
Barbosa 2018[51]	BioMérieux, France	Brazil	Lab assessed	CKD patients from 3 haemodialysis clinics (4.5% HBsAg positive)	286	serum	100% (75.3%–100%);	100% (98.7%–100%)
						whole blood (venepuncture)	92.3% (63.9%–99.8%)	99.6% (97.9%–99.9%)
Dembele 2020[39]	BioMérieux, France	Abidjan, Ivory Coast	Lab	Plasma sample from biobank and whole blood sample from blood donors	1104	Plasma, whole blood	100% (99.2%–100%)	100% (99.2%–100%)
SD Bioline HBsAg rapid test (WHO PQ listed in December 2017; can be stored at up to 40 degree[30])								
Upreti 2014[48]	Standard Diagnostics, Inc. Korea	Nepal	Field	Children and mother (of children with positive field-based tests)	347	serum	100% (68%–100%)	100% (99%–100%)
Sullivan 2019[49]	Standard Diagnostics, Inc	North Territory, Australia	Field	Indigenous Australians in 4 remote communities; additional HBsAg positive participants identified from database	155	serum	91.7% (73%–99%)	100% (97.2%–100%)
Kabamba 2020 [50]	Standard Diagnostics, Inc, Korea	Lubumbashi, Democratic Republic of Congo	Lab	Serum sample from blood donors (100 HBV+)	200	serum	100% (97.7%–100%)	97.1% (96.4%–100%)
Dembele 2020[39]	Standard Diagnostics, Inc, Korea	Abidjan, Ivory Coast	Lab	Plasma sample from biobank and whole blood sample from blood donors	1104	Plasma, whole blood	99.5% (98.3%–99.9%)	99.8% (98.9%–100%)
